# Cooperative and divergent properties of bacterial actin isoforms in *Spiroplasma* swimming

**DOI:** 10.2142/biophysico.bppb-v23.0012

**Published:** 2026-03-12

**Authors:** Daichi Takahashi, Makoto Miyata, Ikuko Fujiwara

**Affiliations:** 1 Research Institute for Interdisciplinary Science, Okayama University, Okayama 700-8530, Japan; 2 Graduate School of Science, Osaka Metropolitan University, Osaka 558-8585, Japan; 3 The OMU Center for Advanced Research for Integrated Natural Science and Technology (OCARINA), Osaka Metropolitan University, Osaka 558-8585, Japan; 4 Department of Materials Sciences and Bioengineering, Nagaoka University of Technology, Nagaoka, Niigata 940-2188, Japan

**Keywords:** actin superfamily, bacterial cytoskeleton, cytoskeletal crosstalk, *Mycoplasma*, Mollicutes

## Abstract

The cytoskeleton, comprising intracellular filamentous structures composed of polymerized proteins, is crucial for the survival of both eukaryotes and prokaryotes. Although bacterial cytoskeletal proteins have diverged, they generally do not drive cellular motility. *Spiroplasma*, a genus of wall-less helical bacteria, swims by propagating a helicity-switching point (kink) along its cell axis. Unlike typical walled bacteria, whose motility depends on widespread motility machineries such as flagella and pili, *Spiroplasma* swimming is powered by the coordinated dynamics of five isoforms of bacterial actin MreB (SMreB1–5), which are grouped into three phylogenetic classes: SMreB1 and 4, SMreB2 and 5, and SMreB3. Despite the efforts to understand *Spiroplasma* swimming, its molecular mechanism remains unclear. In this review, we summarize how *in vitro* analyses of SMreBs have provided mechanistic insights into *Spiroplasma* swimming. While all SMreBs conserve the canonical actin fold, each SMreB class exhibits unique characteristics in its polymerized structures, ATPase activities, polymerization dynamics, and membrane binding. Studies of an essential SMreB subset for *Spiroplasma* swimming, i.e. SMreB1 and SMreB5, have revealed that SMreB1 binds to polymerized SMreB5 and disassembles it depending on the nucleotide state. These results challenge the previous model in which *Spiroplasma* swimming is driven by the coordinated extension and contraction of two distinct SMreB filaments. Finally, we discuss potential molecular mechanisms underlying *Spiroplasma* swimming and highlight key questions that must be answered to validate these models.

## Significance

*Spiroplasma* swimming is the sole bacterial motility system driven by the dynamics of bacterial cytoskeletal proteins. Since the swimming force is generated by a combination of just two isoforms of bacterial actin proteins (SMreBs), *Spiroplasma* swimming is considered one of the simplest motility systems found in organisms on Earth. Therefore, understanding the mechanism of *Spiroplasma* swimming is important not only for understanding the survival strategy of *Spiroplasma*, but also for developing self-propelling nanomachines. This review summarizes the molecular properties of SMreBs revealed by biochemical and structural studies and suggests potential molecular mechanisms of *Spiroplasma* swimming.

## Introduction

The cytoskeleton comprises multiple intracellular filamentous systems composed of polymerized proteins and is indispensable for the survival in all three domains of life: Eukarya, Bacteria, and Archaea. Actin and tubulin superfamily proteins, in particular, are crucial for regulating cellular dynamics [1]. In eukaryotes, not only are actin and tubulin genes well conserved, but their amino acid sequences are also highly conserved compared with those of other essential proteins (the sequence identities of human and yeast actins and tubulins are up to approximately 90% and 75%, respectively) [2,3]. These well-conserved cytoskeletal proteins orchestrate with countless binding proteins to perform various cellular functions, including motility, cytokinesis, and morphogenesis [4–9].

**Figure 1:**
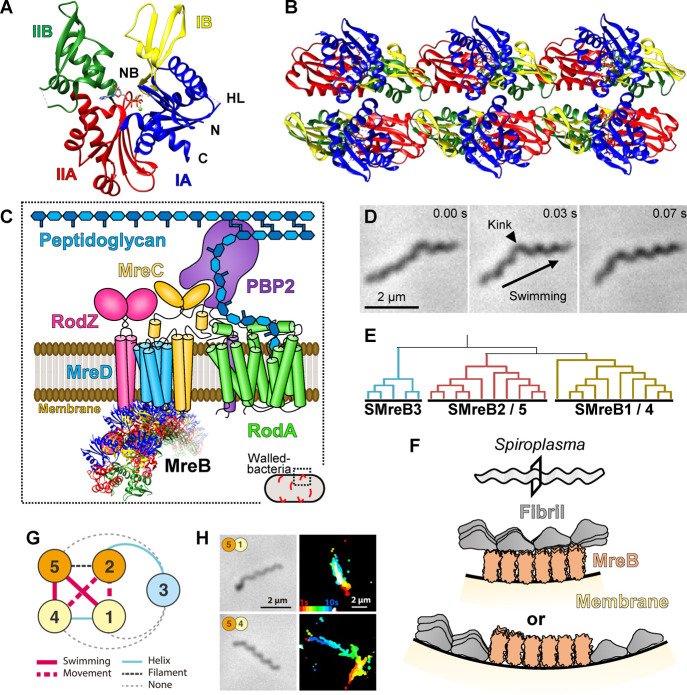
Characteristics of *Spiroplasma* swimming and MreB proteins. (A–B) Ribbon representation of the monomeric (A) and antiparallel double-stranded filament (B) of *Caulobacter crescentus* MreB (CcMreB) (PDB: 4CZJ). Subdomains IA, IB, IIA, and IIB are colored blue, yellow, red, and green, respectively, and labeled in panel A. Panel A also indicates the positions of the N- and C-termini, the nucleotide binding pocket, and the hydrophobic loop for the membrane binding with labels “N”, “C”, “NB”, and “HL”, respectively. (C) Schematic image of the elongasome complex. This scheme summarizes the membrane-binding mode of the MreB filament [69], the PBP2 conformation during the peptidoglycan synthesis [70,71], the self-interactions of MreC [72] and RodZ [73], and the interactions between MreB-RodZ [74], RodA-PBP2 [71,75], PBP2-MreC [76], and MreC-MreD [77]. The focusing point within a walled bacterial cell is indicated by a dashed-square in the lower right schematic image of a cell. (D) Time-lapse phase-contrast microscopy images of a swimming *Spiroplasma* cell taken every 0.03 s. The position of the kink and the swimming direction are indicated in the second frame. Bar indicates 2 μm. (E) Scheme of SMreB phylogeny. Lineages for SMreB1 and 4, SMreB2 and 5, and SMreB3 are highlighted in dark yellow, red, and cyan, respectively. (F) A scheme showing the localization of cytoskeletal proteins in *Spiroplasma* cells. (Top) Scheme of a *Spiroplasma* cell. The cross-section focused in the lower schemes is indicated by a square. (Middle and bottom) Models showing the localization of fibril and MreB filaments. One study has reported that an SMreB sheet localizes between the cell membrane and a fibril sheet (middle) [56], whereas another study has reported that fibril sheets localize underneath the cell membrane at the both sides of a membrane-bound SMreB sheet (bottom) [57]. (G–H) The morphology and behavior of syn3B cells expressing two out of the five SMreBs. (G) Summary of syn3B cell morphologies expressing all the possible pairs of two SMreBs. Each SMreB isoform is denoted by a numbered circle. The line style connecting the circles indicates the syn3B cell morphology expressing the corresponding pair of SMreBs; red solid: cells swim directionally, red dashed: cells undulate in place, cyan solid: cells form a static helical shape, black dashed: cells are elongated without helix formation, and gray dashed: cell morphologies are unchanged. (H) Phase-contrast microscopy image (left) and compiled time-lapse images (right), generated by overlaying frames acquired over 10 s, color-coded from red (0 s) to blue (10 s), of swimming syn3B cells expressing SMreB1 and SMreB5 (top) and SMreB4 and SMreB5 (bottom). Bar indicates 2 μm. These figures are modified versions of a previous study [63].

Until the 1990s, it was widely believed that the cytoskeleton was exclusive to eukaryotes and absent in prokaryotes, which are classified as either bacteria or archaea and have been characterized as having small, simple cells. Since then, however, numerous types of prokaryotic cytoskeletal proteins have been discovered, beginning with the identification of MreB and FtsZ, which are bacterial proteins belonging to the actin and tubulin superfamilies, respectively [10–20]. MreB polymerizes into an antiparallel double-stranded filament (Figures 1A and 1B) that serves as a scaffold for forming and localizing the elongasome complex, the bacterial cell-wall (peptidoglycan) synthesis complex responsible for the cell growth (Figure 1C) [21,22]. FtsZ forms a single-stranded polar filament to assemble and localize the divisome complex, the bacterial cell-wall synthesis complex involved in the cell division [23,24]. Templated by these filaments, both proteins built higher-ordered structures, such as MreB sheets and antiparallel double-stranded filaments and toroidal structures of FtsZ [25–30]. Unlike eukaryotic cytoskeletal proteins in which highly conserved actin and tubulin are utilized for numerous phenomena, bacteria have developed several dozens of cytoskeletal proteins for their viability based on the “one filament, one function” theory [3,31,32]. Accordingly, individual bacterial cytoskeletal proteins are typically dedicated to distinct cellular processes. Examples include partitioning low-copy-number plasmids with ParM, AlfA, or TubZ [33–38], anchoring FtsZ filaments to the membrane with FtsA [39,40], and aligning magnetosomes (metal-enriched organelles in magnetotactic bacteria to sense magnetic fields) with MamK [41,42]. Despite this diversity, bacterial cytoskeletal proteins have not been reported to drive cellular motility like eukaryotic ones, except for the actin superfamily proteins in the genus *Spiroplasma*.

*Spiroplasma*, pathogenic for plants and arthropods, has a wall-less helical cell with the cell length of 2–10 μm [43,44]. It swims in viscous liquids by propagating a cell-helicity switch point (kink) from the cell tip to the end, generating the propulsive force (Figure 1D) [45,46]. This motility is necessary not only to move in an aqueous phase, but also involved in the pathogenicity of *Spiroplasma* [47,48]. To drive this motility, *Spiroplasma* has evolved five MreB isoforms (SMreB1–5) from those involved in the cell-shape maintenance of walled bacteria [49,50]. This phenomenon may reflect the evolutionary adaptation of *Sprioplasma* to acquire a motility system in cells lacking a peptidoglycan layer, which is necessary scaffold for driving conventional bacterial motility machineries, such as flagella and pili [51,52]. The unique swimming motility of *Spiroplasma* has been studied from various perspectives, including microscopy [46,53–59], physics-based modeling [45,60,61], and genetics [62–66]. However, despite these studies, the underlying molecular mechanism remains unclear. In this review, we summarize the molecular properties of SMreBs as clarified by *in vitro* analyses, including our own, and discuss how *Spiroplasma* swimming is driven at the molecular level. We also address the underlying questions in this field.

## Unique swimming machinery of *Spiroplasma* composed of bacterial actin proteins

The SMreBs form an intracellular ribbon structure for driving *Sprioplasma* swimming in cooperation with a fibril, which is a cytoskeletal protein unique to *Spiroplasma* [56,57]. The ribbon structure consists of a dumbbell structure at its tip and the following sheet structure along the innermost line of the helical *Spiroplasma* cell [67]. There are conflicting reports on the architecture of the sheet structure, which is important for propagating the kink (Figure 1D). When the *Spiroplasma* cell body is examined in a transverse section (Figure 1F), one paper has reported that the sheet structure is composed of an SMreB sheet sandwiched between a fibril sheet and the cell membrane (Figure 1F middle) [56]. In contrast, another paper reported that an SMreB sheet, accompanied by fibril sheets on both sides, localizes underneath the cell membrane in the sheet region (Figure 1F bottom) [57]. Although the relationship between these localization patterns remains controversial, both models agree on the position of the SMreB sheet in relation to the cell membrane. It is important to note that neither study identified the exact SMreB isoform that constructs the ribbon structure, and they might have overlooked dynamic structures that continuously associate with and dissociate from the ribbon due to the observation of fixed cells. Additionally, it remains unclear which dynamic processes within the ribbon structure are responsible for driving *Spiroplasma* swimming.

On the other hand, recent advances in genetic research are clarifying the roles of SMreBs. Gene manipulation of *Spiroplasma* has been considered difficult due to the limited number of available antibiotics for use as the selective markers and the substantial decrease in growth efficiency during gene manipulation procedures [66,68]. However, these difficulties have been overcome by expressing the cytoskeletal proteins of *Spiroplasma* heterogeneously and reconstituting *Spiroplasma* swimming in *Mycoplasma* (derived) cells easy for the gene manipulation [62,63]. A study using JCVI-syn3B, which carries the minimum genome essential for the self-replication designed from *Mycoplasma mycoides* [78,79], revealed that the combinations of two SMreB isoforms, either SMreB1 or 4 with either SMreB2 or 5, are sufficient for the force generation for motility, and fibril and SMreB3 are not essential for its process (Figure 1G). Especially, co-expression of SMreB5 with either SMreB1 or 4 reconstructed the kink propagation and the swimming motility (Figure 1H) [63]. The essentiality of SMreB5 has also been demonstrated by another heterogenous expression system and an analysis of a naturally isolated *Spiroplasma* mutant [62,65]. Several reports also indicate that MreBs in walled bacteria are involved in various motility systems, such as adventurous motility (A-motility) of a soil bacterium *Myxococcus xanthus* and comet-tail motility of *Shigella* hijacking the actin filaments in a host cell [80,81]. However, these MreBs are involved in scaffolding and localizing the motility machineries rather than being incorporated into the core force-generation units of these motilities. Therefore, *Spiroplasma* swimming is currently believed to be the sole motility system driven by the dynamics of bacterial cytoskeletal proteins.

As described in the motility reconstitution experiments above, sequence identity analysis also groups the five SMreB isoforms into three classes: the classes of SMreB1 and 4, SMreB2 and 5, and SMreB3 (Figure 1E) [64]. Although amino acid sequence identities are up to approximately 70% between SMreB1 and 4 and approximately 60% between SMreB2 and 5, those between the other classes remain about 40% [49,65]. In this context, *Spiroplasma* swimming, which is driven by the combinations of SMreB5 and either SMreB1 or 4, can be interpreted as a motility system involving only two SMreB classes. The number of factors, up to two classes, essential for *Spiroplasma* swimming is remarkably small in the prokaryotic motility systems which often rely on huge, complex protein machineries [51,82–86]. In eukaryotes, actin-based motility may be recalled as a motility system requiring a small number of factors. However, this system requires at least five factors, including actin itself; the Arp2/3 complex for branching actin filaments; its activation factor localizing underneath the membrane (like the WAVE regulatory complex); actin depolymerizing factor (ADF)-cofilin for severing actin filaments; and a capping protein for inhibiting the spontaneous nucleation of actin filaments [87]. Therefore, *Spiroplasma* swimming, driven by only two proteins, can be considered one of the simplest motility systems on Earth.

## Characteristics of SMreBs revealed by *in vitro* studies

*In vitro* biochemical assays and structural analyses are powerful approaches for characterizing motility factors. Recently, several groups including us have extensively studied SMreBs, clarifying their properties from various aspects. In this section, we will summarize the molecular properties of the SMreBs, with a focus on their biochemistry and structural biology, underlying *Spiroplasma* swimming.

## Polymerized structures

**Figure 2:**
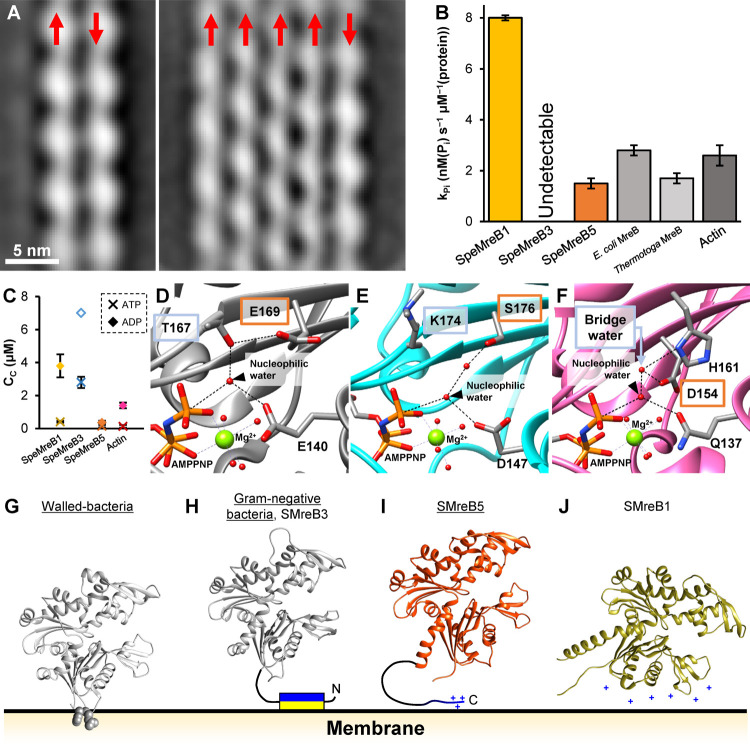
Characteristics of SMreBs revealed by biochemical and structural studies. (A) Two-dimensionally averaged images of an SpeMreB5 filament (left) and an SpeMreB5 sheet composed of five protofilaments (right) observed by negative staining electron microscopy. The polarity of each protofilament is indicated by an arrow at the top. Bar indicates 5 nm. This figure is a modified version of our previous paper [91]. (B) P_i_ release rates of MreB family proteins and eukaryotic actin summarized from the literatures [29,88,91,108,109]. Bars indicate the standard deviation reported in each literature. (C) Critical concentrations of SpeMreB1, SpeMreB3, SpeMreB5, and rabbit skeletal actin in the presence of ATP (crosses) and ADP (diamonds), summarized from the literatures [88,91,110]. Bars indicate the standard deviation reported in each literature. The critical concentration of SpeMreB3 in the presence of ADP, which was estimated as an approximated value due to the small precipitation amounts, is indicated by an open symbol without bars. (D–F) Close-up views of the active sites for ATP hydrolysis in (D) a typical MreB family protein represented by CcMreB (PDB: 4CZJ), (E) SpeMreB3 with chemically di-methylated lysine residues (PDB: 7E1G), and (F) a eukaryotic F-actin (PDB: 7W4Z) all in the Mg^2+^-AMPPNP bound-state. Water molecules are indicated by red spheres. The residues or water corresponding to the catalytic core for ATP hydrolysis are highlighted in pale blue and orange. These figures are modified versions of our previous paper [91]. (G–J) Membrane binding modes of (G) MreB in walled bacteria (PDB: 1JCF), (H) MreB in several Gram-negative bacteria and SMreB3 (PDB: 4CZM) (the N-terminal amphipathic helix is indicated by a rectangle in which the hydrophobic and hydrophilic surfaces are colored yellow and blue, respectively), (I) SMreB5 (PDB: 7BVY), and (J) SMreB1 (full-length SpeMreB1 structure modeled by AlphaFold 2 [111]). Labels with an underbar indicate that both the affinity for the membrane and the membrane-binding region have been evaluated experimentally.

SMreB1, SMreB3, and SMreB5 have been expressed in *E. coli*, purified, and analyzed for their structures and activities. All of these SMreBs polymerize induced by the nucleotide binding [65,88–92]. SMreB3 and SMreB5 form antiparallel-double stranded filaments, a characteristic also seen in walled bacterial MreBs (Figure 2A left). Additionally, SMreB5 assembles into sheet structures in which protofilaments (units of longitudinally aligned monomers) align parallel to one side of the antiparallel double-stranded filament, confirmed by SMreB5 of a crustacean pathogen *Spiroplasma eriocheiris* (SpeMreB5) (Figure 2A right) [91]. This protofilament alignment makes the overall sheet structure asymmetric along the major axis, which is defined by the direction of the filament elongation, thereby meeting a requirement for driving a directed motility like *Spiroplasma* swimming. Above a certain protein concentration, SMreB5 further assembles into bundle structures depending on the ionic strength, divalent cations, and its ATPase activity [89,92]. Sheet and bundle formations have also been confirmed in SMreB1, although its structure and molecular properties have not been analyzed as thoroughly as those of SMreB5 [88].

## ATPase and polymerization activities

In eukaryotic actin, the ATPase reaction cycle is tightly coupled to its polymerization dynamics [93,94]. Although ATP hydrolysis is negligible in the monomeric G-actin, it is accelerated by several tens of thousands-fold when shifting to the polymerized F-actin [95]. ATP is hydrolyzed immediately after the actin polymerization at a rate constant of 0.3 s^–1^ [96], whereas the release of an inorganic phosphate (P_i_) is slow with a half-life of about six minutes to shift from the ADP-P_i_ state to the ADP-state [97]. ADP-P_i_ bound actin subunits stabilize the filaments, whereas ADP-bound subunits dissociate relatively rapidly from the filament ends, linking the nucleotide state to actin turnover [97–101]. This nucleotide-dependent affinity of actin is not only important for filament assembly and disassembly but also for its interactions with a wide range of actin-binding proteins [102–104]. ATPase activities have also been confirmed in many MreB family proteins including SMreBs (Figure 2B) [29,30,88,90,91,105–108], although their biological roles remain controversial. Intriguingly, the ATPase activities differ dramatically among the SMreBs. Based on the P_i_ release rates as the indicative of the ATPase activity, SMreB5 shows the rate relative to those of other MreB family proteins and skeletal actin [29,91,104–109]. Surprisingly, the P_i_ release rate of SMreB1 is approximately five-fold faster than that of SMreB5, demonstrating the fastest rate among the MreB family proteins [88]. In contrast, the rate of SMreB3 falls below the detection limit of a conventional method for measuring P_i_ release [91]. These results indicate that SMreBs exhibit entirely distinct ATPase activities across each class.

Understanding the polymerization dynamics of cytoskeletal proteins requires evaluating not only ATPase activities, but also how these activities are coupled to filament assembly and disassembly. Although the polymerization procedures of SMreBs are not directly observed, their polymerization properties are evaluated using critical concentration measurements under steady state conditions (Figure 2C) [88,89,91]. The critical concentration is the protein concentration at which the association rate exceeds the dissociation rate, i.e., the minimum concentration required for the polymerization [1]. The critical concentrations of SMreB1 and SMreB5 in the presence of ATP correspond to those of eukaryotic actin and other MreB family proteins [88,106,107,112,113]. The critical concentrations of SMreB5 in the presence of ADP or AMPPNP (a nonhydrolyzable ATP-analog) are only two- to three-fold higher than that in the presence of ATP, which is similar to those of other MreB family proteins [91,106,107]. In contrast, SMreB1 exhibits a critical concentration approximately nine-fold higher in the presence of ADP than that in the presence of ATP and rarely polymerizes in the presence of AMPPNP [88]. Furthermore, the critical concentration of SMreB1 in the presence of ADP is 2.7 times higher than that of eukaryotic actin [110]. These results indicate that SMreB1 is stabilized in the polymerization state after the ATP hydrolysis and depolymerizes immediately after P_i_ release, whereas SMreB5 tends to retain its polymerized state throughout the ATPase reaction cycle. Given the fast P_i_ release rate of SMreB1 [88], its filaments are likely to undergo frequent subunit exchange. In contrast, SMreB5 filaments appear to be static, as was also suggested for walled bacterial MreBs [114,115].

Conversely, SMreB3 polymerizes in the presence of ATP, albeit at a critical concentration about ten-times higher than that of SMreB5. However, SMreB3 rarely polymerizes in the presence of ADP or AMPPNP [91] (Figure 2C). These results suggest that SMreB3 stabilizes its polymerized state through its ATP hydrolysis, albeit slowly, and exchanges its subunits in the filaments at an especially slow rate, even among MreB family proteins, whose polymerization dynamics is supposed to be slow in the actin superfamily proteins [114,115].

## ATP hydrolysis mechanism

As summarized in the previous section, there are differences in ATPase activity among SMreBs (Figure 2B). The low ATPase activity of SMreB3 can be explained by the structural differences in the active site for ATP hydrolysis among actin superfamily proteins (NB in Figure 1A and Figure 2D–F) [91]. The detailed mechanism of ATP hydrolysis in F-actin has been clarified based on the atomic-resolution structures and quantum mechanics simulations, where the reaction proceeds with a proton elimination from the nucleophilic water by a bridge water conjugated with the D154 residue, followed by the nucleophilic attack of the nucleophilic water to the γ-P_i_ and a proton transfer from the bridge water to an oxygen atom in the γ-P_i_ (Figure 2F) [116]. The atomic positions in the F-actin active site is conserved in that of most walled-bacterial MreBs, as seen in *Caulobacter crescentus* MreB (CcMreB) (the bridge water and D154 in actin correspond to T167 and E169, respectively, in CcMreB) (Figure 2D) [22]. This structural similarity suggests that the ATP hydrolysis mechanism of F-actin is common to those of the MreB family proteins and is consistent with the fact that the P_i_ release rates of many MreB family proteins are in line with that of eukaryotic actin (Figure 2B). However, the residues corresponding to T167 and E169 in CcMreB are substituted with K174 and S176 in SMreB3 and do not form a hydrogen-bonding network with the nucleophilic water (Figure 2E) [91]. Consequently, SMreB3 is probably unable to transfer a proton from the nucleophilic water to catalyze ATP hydrolysis, resulting in the slow P_i_ release.

Unlike SMreB3, which has a distinct active site structure compared to the MreB family proteins, SMreB1 has the active site residues common to the MreB family proteins including CcMreB and SMreB5 [49], suggesting that a factor other than ATP hydrolysis is involved in the fast P_i_ release rate of SMreB1.

## Membrane binding mode

MreB family proteins are well known to work beneath the cell membrane [21,56,57]. Most MreBs of walled-bacteria bind to the membrane through two consecutive hydrophobic amino acid residues in the “hydrophobic loop” within subdomain IA (HL in Figure 1A and Figure 2G) of four subdomains in an MreB molecule (subdomains IA, IB, IIA, and IIB) (Figure 1A). MreBs of several Gram-negative bacteria additionally equip an amphipathic helix at the N-termini for the membrane binding (Figure 2H) [69]. Sequence analysis shows that SMreB2, 3, and 5 retain hydrophobic residues in the hydrophobic loop, whereas an N-terminal amphipathic helix is present only in SMreB3 (Figure 2H). By contrast, SMreB1 and 4 lack these canonical membrane-binding motifs and were therefore not expected to associate with membranes [49]. However, membrane binding assays have confirmed that SMreBs have acquired unique membrane binding modes. For SMreB5, the unstructured, positively charged C-terminal region is essential for the membrane binding rather than the hydrophobic residues in the hydrophobic loop (Figure 2I) [92]. This feature likely reflects evolutionary adaptation to the *Spiroplasma* membrane, which is enriched in negatively charged lipids [117]. Contrary to the expectation based on the amino acid sequences, SMreB1 also binds to the membranes, mimicking the lipid composition of a *Spiroplasma* membrane [88]. Although its precise membrane-binding region has not been identified, the extended positively charged surface within the subdomain IA of SMreB1 suggests an orientation at the membrane comparable to that of other MreB family proteins (Figure 2J). Together, SMreB1 and 5 have acquired the unique membrane-binding modes that are not found in MreBs from walled bacteria, likely reflecting their specialized roles in driving *Spiroplasma* swimming.

## Crosstalk of SMreB1 and 5, an essential set for *Spiroplasma* swimming

**Figure 3:**
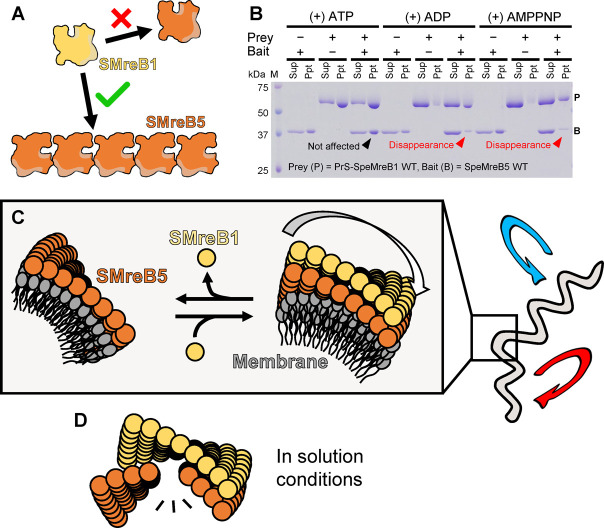
Crosstalk of SMreB1 and 5, an essential set for *Spiroplasma* swimming. (A) Binding mode of SMreB1 to SMreB5. SMreB1 binds to polymerized SMreB5, but not to SMreB5 monomers. This figure is a modified version of our previous study [88]. (B) Co-sedimentation assays of SpeMreB1 fused with a solubilization-tag, proteinS (PrS) [121], and SpeMreB5. SpeMreB5 precipitation disappears in the presence of PrS-SpeMreB1 in the ATP-state-dependent manner. This figure is a modified version of our previous study [88]. (C) A perspective on the helicity switching mechanism for *Spiroplasma* swimming. The conformation of the SMreB5 sheet underneath the cell membrane shifts to switch the cell helicity when SMreB1 that continuously polymerizes on and dissociates from the SMreB5 sheet. (D) A perspective on the destabilization mechanism of polymerized SMreB5 by SMreB1 in solution conditions.

Since co-expression of either SMreB1 or 4 with either SMreB2 or 5 is required for the *Spiroplasma* motility reconstruction into JCVI-syn3B [63], the crosstalk of these SMreBs is probably crucial for force generation. A study using a heterologous expression system of SMreBs in *E. coli* cells indicates that SMreB1 inhibits the filament formation of SMreB2 [64]. We examined the crosstalk between SMreB1 and 5 using co-sedimentation assays with an ultracentrifugation [88]. Combined analyses of these polymerization-deficient variants reveal that the SMreB5 polymerization is necessary for the binding between SMreB1 and 5 (Figure 3A). Additionally, we demonstrate that SMreB1 destabilizes the polymerized SMreB5 depending on the nucleotide state (Figure 3B). In the presence of ADP or AMPPNP, twice the concentration of SMreB1 eliminates SMreB5 from the precipitated fraction in co-sedimentation assays. This result indicates enhanced destabilization of SMreB5 filaments by SMreB1. Although this phenomenon is confirmed in the polymerization-deficient variant of SMreB1, its polymerization is actually essential for *Spiroplasma* swimming [88]. These results suggest that a continuous crosstalk between SMreB1 and 5 is crucial for *Spiroplasma* swimming.

## Perspectives of the molecular mechanism of *Spiroplasmsa* swimming

Based on the above findings, we discuss the possible molecular mechanism of *Spiroplasma* swimming. Given that the pair of SMreB1 and 5 is one of the minimum sets necessary for the motility [63], a central question is how SMreB1 destabilizes the SMreB5 sheet. SMreB1 does not bind to the SMreB5 monomers in any nucleotide state. Additionally, polymerization-deficient variants of SMreB1 did not destabilize the SMreB5 sheets in the presence of ATP [88]. These observations suggest that SMreB1 does not inhibit SMreB5 polymerization through monomer sequestration or filament-end capping. Rather, the data are most consistent with a model in which SMreB1 affects preassembled SMreB5 structures. One plausible scenario is that SMreB1 locally destabilizes the assembled structure of SMreB5 and alters the SMreB composition within the ribbon structure, leading to a switch in cell helicity. Considering that SMreB1 destabilizes the SMreB5 sheets depending on the nucleotide state, this model assumes that SMreB1 dissociates SMreB5 upon the P_i_ release of SMreB5. Subsequently, the cell helicity is restored with the P_i_ release from SMreB1 and the SMreB5 re-polymerization. In this scenario, the differences in the P_i_ release rates between SMreB1 and 5 correspond to the differences in cell helicity switching from right to left and *vice versa*. However, about the five-fold difference in the P_i_ release rates between SMreB1 and 5 [88] is inconsistent with the estimated differences of the helicity switching rates from right- to left-handed and *vice versa* in *Spiroplasma* cells, as determined by optical microscopy [46,54]. Therefore, although experiments under solution conditions appear to show SMreB1 dissociating SMreB5 sheets, SMreB1 may induce a conformational change in the SMreB5 sheets to switch the cell helicity. Based on the morphological changes caused by SMreB5 expression in *Mycoplasma mycoides* cells, the SMreB5 sheet likely localizes underneath the cell membrane as a “backbone” for the helical cell shape (Figure 3C) [62]. The association and dissociation of SMreB1 with fast polymerization dynamics would then switch the cell helicity like an “actuator” to remodel the SMreB5 sheet conformation. Although SMreB1 dissociates SMreB5 sheets under the solution conditions, the SMreB5 sheet may undergoes a conformational change rather than completely dissociated, supported by various factors such as the cell membrane (Figure 3D). This analogy, in which SMreB5 sheets are dissociated in solution due to SMreB1 but not in the cell, is similar to the severing of sheets or bundles composed of actin filaments by a high concentration of myosin [118,119], which is known to convert the chemical energy of ATP hydrolysis to the mechanical energy to walk along actin filaments or move actin filaments. For this model to be valid, the conformational change of the SMreB5 sheet must occur consecutively from the cell tip to the end. This function could be served by a symmetry-breaking structure, such as the dumbbell structure and/or the asymmetric structure of the SMreB5 sheet [67,91]. This model is not definitive and must be refined in future studies.

A previous paper has reported the “bimetallic strip model” as the molecular mechanism of *Spiroplasma* swimming [120]. In this model, a kink propagates through cooperative switching of the polymerization states of two types of SMreB isoforms. Specifically, the kink propagates through the polymerization of either SMreB1 or 5 and the simultaneous depolymerization of the other SMreB isoform aligned laterally. However, given the differences in biochemical parameters such as P_i_ release rates and critical concentrations depending on the nucleotide states between SMreB1 and 5, it is reasonable to assume that these SMreBs have different cellular functions rather than equivalent roles. In addition, the bimetallic strip model proposes that a depolymerized SMreB subunit retains binding to the filament of the other SMreB isoform within the ribbon structure. However, this contradicts the results showing that SMreB1 does not bind to the SMreB5 monomers (Figure 3A). Therefore, although there is still insufficient data to determine the molecular model of *Spiroplasma* swimming, the bimetallic strip model should be reconsidered.

## Existing questions to clarify the molecular mechanism of *Spiroplasma* swimming

Although we have proposed a molecular model of *Spiroplasma* swimming, there are still many questions that need to be answered. In this section, we summarize the underlying questions about *Spiroplasma* swimming that need to be resolved. We hope that our proposed model will be reinforced and/or improved by solving these kinds of questions.

## Direct observation of the SMreB dynamics

One critical solution for evaluating the above model of *Spiroplasma* swimming is directly observing the polymerization dynamics of SMreB1 and 5 and their interactions. Total internal reflection fluorescence microscopy (TIRFM) has clarified the polymerization kinetics of eukaryotic actin and its cooperation with actin-binding proteins and small molecules by visualizing the dynamics of actin filaments [98,122–125]. Lysine and cysteine residues are widely used as targets for fluorescent labeling; however, the chemical modification of lysine residues on SMreB inhibits its polymerization [91], and there are no cysteine residues on the surfaces of SMreBs possibly available for fluorescent labeling, like C374 on skeletal-actin. Additionally, electron microscopy has shown that SMreB filaments and sheets are often several hundred nanometers long [88,89,91], which is shorter than the spatial resolution of TIRFM. Overcoming these difficulties and enabling direct observation of the SMreB polymerization dynamics would substantially advance our understanding of the molecular mechanisms of *Spiroplasma* swimming.

## Structural analysis of the force generation unit

A structural analysis of the SMreB1 and 5 complex is also necessary for understanding the molecular mechanism of *Spiroplasma* swimming. Structural analyses of MreB family proteins have been proceeded using X-ray crystallography, from its complex with a binding protein to the antiparallel double-stranded filament structure [10,22,65,74,91,92,105]. This type of analysis is likely possible due to an intrinsic property of MreB filaments, which resolve their helicity by binding to a surface [126], enabling to form crystals as an array of straight protofilaments. However, the alignments of protofilaments in MreB crystals do not necessarily correspond to those of MreB filaments and sheets formed in solutions and cells. Therefore, cryoEM, which has determined the structures of numerous proteins complexed with cytoskeletal filaments, will be advantageous for determining the complex structure of SMreB1 and 5. Several studies have observed MreB filaments using cryoEM [22,65,92]; however, these attempts did not lead to their structural determination possibly due to the filament bundling and/or preferred orientation problems. Overcoming these issues to determine the structure of the force generation unit of *Spiroplasma* swimming is also one of the central challenges in this field.

## *In situ* structure of the cytoskeletal proteins linked to the cellular helicity

Several studies have determined the ribbon structure in *Spiroplasma* cells [56,57]. However, the correlation between the cell helicity and the conformational changes in the ribbon structure has not yet been reported. Swimming *Spiroplasma* cells are classified into three regions based on the cellular helicity: right-handed, left-handed, and kinked regions. Analyzing the correlation between the helicity state of *Spiroplasma* cells and the ribbon structure could help develop a more sophisticated swimming model.

## Morphology and behavior of *Spiroplasma* cells carrying SMreB mutations

Analyzing the morphology and cellular dynamics after inducing gene mutations is one of the central approaches for understanding protein functions. Although the genetics of *Spiroplasma* has recognized to be difficult, as discussed in the previous section, the recent approach of reconstituting *Spiroplasma* swimming in other cells also enables the manipulation of the genes coding SMreBs [62,63]. This technology allows us to analyze the effect of SMreB mutations on *Spiroplasma* swimming.

## Role of fibril and SMreB3

Advances in genetics research have revealed that fibril is not essential for *Spiroplasma* swimming [62,63]. However, fibril was considered the motor for *Spiroplasma* swimming until these genetics researches [57,58,127], owing to its abundance in the *Spiroplasma* proteome and the fraction containing the ribbon structure [67,128], suggesting some roles of fibril on *Spiroplasma* swimming. Since fibril makes *Mycoplasma* (derived) cells a static helix and exhibit no obvious enzymatic activity or polymerization dynamics [53,62,63], its role may be to mechanically support the cells, similar to the well-established mechanical functions of intermediate filaments [129,130]. Additionally, although SMreB3 was also concluded to be unnecessary for *Spiroplasma* swimming [62,63], its gene is conserved with a hypothetical lipoprotein immediately upstream of the operon coding SMreB4 and 5 in approximately half of the identified *Spiroplasma* species [131]. Understanding the supportive functions of fibril and SMreB3 will emphasize the understanding of the essential roles of other SMreBs that directly drive *Spiroplasma* swimming.

## Factor(s) to accelerate SMreB dynamics

*In vitro* analysis has suggested that SMreB5 acts as the “backbone” of the helical cell shape, whereas SMreB1 acts as an “actuator” that changes the conformation of the SMreB5 sheet to drive *Spiroplasma* swimming. However, there is a substantial discrepancy between the activities estimated from biochemistry and the rates of the cellular dynamics. If the cell helicity switch is governed by the conformational change of an SMreB5 sheet driven by the polymerization dynamics of SMreB1, as was suggested by the above model, then the kink propagation rate and kink generation frequency would correspond to the polymerization and depolymerization rates of SMreB1, respectively. Assuming that the subunit length and polymerization rate of SMreB1 are the same as those of actin (i.e., 5 nm and 11.6 μM^–1^ s^–1^, respectively [113,132–134]), the estimated kink propagation rate of *Spiroplasma* cells (about 10 μm s^–1^ [46,54]) could be achieved with a cellular SMreB1 concentration of about 200 μM. This assumption is likely reasonable, considering that the actin concentration in a vertebrate cell is up to 150–200 μM [135,136], and the intracellular concentrations of SMreB1 and 4 are so high that the appearance of mRNA for their translation accounts for top 10% of the *Spiroplasma* transcriptome [64]. However, if we assume that the P_i_ release rate of SMreB1 corresponds to its depolymerization rate, then the residence time of the helicity switching is estimated to be about two minutes. This is contradictory to the microscopy results, which show that a kink is generated at the cell tip when the leading kink is about to reach the cell end [46,54]. Although the *in vitro* activity of SMreB1 has been measured as the fusion with a solubilization tag [88,121] possibly decreasing the apparent activities, its effect is possibly limited. Therefore, there may be several factors accelerating the SMreB dynamics in cells.

## Conclusions

In this review, we have summarized the molecular features of SMreBs involved in *Spiroplasma* swimming, which is one of the simplest motility systems on Earth. Recent *in vitro* analyses, including our biochemical and structural studies, have clarified that *Spiroplasma* utilizes SMreBs with completely different molecular properties to enable its distinctive swimming motility. The next challenge in this field is to understand in detail how SMreBs cooperate with each other to drive *Spiroplasma* swimming. Understanding its molecular mechanism will not only unveil the survival strategy of *Spiroplasma*, but also will help us understand its evolutionary history, construct the technology to suppress the *Spiroplasma* pathogenicity, and develop a self-propelling nanomachine.

## Conflict of interest

Authors declare that they have no competing interests.

## Author contributions

The original draft has been written by DT and then reviewed and edited by the all authors.

## Data availability

The evidence data generated and/or analyzed during the current study are available from the corresponding author on reasonable request.
